# Using convolutional neural network denoising to reduce ambiguity in X-ray coherent diffraction imaging

**DOI:** 10.1107/S1600577524006519

**Published:** 2024-08-05

**Authors:** Kang-Ching Chu, Chia-Hui Yeh, Jhih-Min Lin, Chun-Yu Chen, Chi-Yuan Cheng, Yi-Qi Yeh, Yu-Shan Huang, Yi-Wei Tsai

**Affiliations:** a National Synchrotron Radiation Research Center Hsinchu300 Taiwan; b Department of Physics National Tsing Hua University,Hsinchu300 Taiwan; Paul Scherrer Institut, Switzerland

**Keywords:** coherent diffraction imaging, machine learning, Noise2Noise, mixed-scale dense network

## Abstract

The inherent ambiguity in reconstructed images from coherent diffraction imaging (CDI) poses an intrinsic challenge, as images derived from the same dataset under varying initial conditions often display inconsistencies. This study introduces a method that employs the Noise2Noise approach combined with neural networks to effectively mitigate these ambiguities. We applied this methodology to hundreds of ambiguous reconstructed images retrieved from a single diffraction pattern using a conventional retrieval algorithm. Our results demonstrate that ambiguous features in these reconstructions are effectively treated as inter-reconstruction noise and are significantly reduced. The post-Noise2Noise treated images closely approximate the average and singular value decomposition analysis of various reconstructions, providing consistent and reliable reconstructions.

## Abstract 2

Ambiguity, which is an intrinsic characteristic of coherent diffraction imaging during image retrieval, can be reduced using machine learning.

## 1. Introduction

Coherent diffraction imaging (CDI), an oversampling based lensless imaging technique, is realized by capturing coherent scattering patterns and applying retrieval algorithms to reconstruct images. To facilitate CDI, obtaining high-coherence light sources, particularly in the hard X-ray region, is challenging. At the end of the twentieth century, the emergence of partially coherent X-rays from third-generation synchrotron radiation facilities advanced X-ray CDI. Subsequently, free-electron laser facilities emitting fully coherent X-rays further developed these techniques. Recently, fourth-generation synchrotron radiation facilities near diffraction-limited X-ray sources have increased the prevalence of CDI and related lensless imaging techniques.

These lensless imaging techniques have intrinsic limitations. In the early stages, to satisfy the oversampling method criteria in CDI (Sayre, 1980 ▸; Miao *et al.*, 1999 ▸), the sample size had to be isolated and smaller than the illuminated beam size. Recently, an increasing number of CDI based lensless X-ray imaging techniques have been proposed to overcome the limitations of CDI. For instance, Kang *et al.* (2021 ▸) and Takazawa *et al.* (2021 ▸) used apertures that were larger than the sample to define the sample profile and to overcome the beam size limitation in CDI. Takayama *et al.* (2021 ▸) collected a series of coherent scattering patterns over time to achieve time-resolved CDI. Vartanyants & Robinson (2003 ▸) applied a partially coherent beam to a structured quantum-dot array to observe the average shape and orientation of an individual island. Ayyer *et al.* (2016 ▸) demonstrated that the loss correlation of an array containing identical objects yields diffraction peaks from the array and scattering patterns from individual objects. Chen *et al.* (2023 ▸) proposed an ensemble CDI method using numerous randomly arranged identical unit patterns to enhance the signal-to-noise ratio of a diffraction pattern. Furthermore, the methods for improving the robustness and resolution of CDI are also reported. Sekiguchi *et al.* (2017 ▸) proposed a protocol to efficiently yield realistic maps with low similarity scores (Yoshida *et al.*, 2024 ▸) that were based on the empirical observation in their prior study. In addition, the similarity score, characterized in terms of the phase differences between the structure factors of the retrieved maps, was proposed by Takayama & Nakasako (2024 ▸).

However, CDI exhibits ambiguity during retrieval, and the retrieved images with different initial conditions are inconsistent. Various guiding methods have been employed to improve the reliability of retrieval algorithms (Chou & Lee, 2002 ▸; Chou *et al.*, 2003 ▸; Chen *et al.*, 2007 ▸). In order to improve robustness, free log-likelihood as an unbiased metric for CDI has been proposed (Favre-Nicolin *et al.*, 2020 ▸). Furthermore, in the same research, the eigen-solution analysis conducted by performing singular value decomposition (SVD) on datasets can successfully reduce ambiguity. Recently, machine learning (ML) showed a significant advantage for solving many computational imaging problems, such as semantic segmentation (Badrinarayanan *et al.*, 2017 ▸; Ronneberger *et al.*, 2015 ▸; Long *et al.*, 2015 ▸) and image classification (Agrawal *et al.*, 2014 ▸; Simonyan & Zisserman, 2015 ▸; He *et al.*, 2015 ▸). In CDI experiments, ML also plays an important role and has been considered revolutionary for image reconstruction. For instance, Wu *et al.* (2021 ▸) and Cherukara *et al.* (2018 ▸) used convolutional neural networks for rapid two-dimensional phase retrieval. In addition, a three-dimensional ML model combining supervised learning with transfer learning to the complex morphological information of a range of nanoparticles has also been proposed (Wu *et al.*, 2021 ▸). Bellisario *et al.* (2022 ▸) used deep learning as a tool to denoise and demask diffraction patterns on simulated images. Image applications have been receiving increasing attention for decades due to progress in computational capabilities.

This study proposes a potential method based on the Noise2Noise approach to denoise CDI images and hence reduce ambiguity. An open-source implementation in Python, corresponding to a mixed-scale dense network architecture, was used. The proposed neural network can efficiently extract reconstruction related consistent features and reduce ambiguity in various reconstructions.

## 2. Experimental

To elucidate the ambiguity inherent in CDI reconstructions, we adapted a measurement using ensemble CDI (eCDI) with a standard sample. For more details on the eCDI measurement, please refer to previous work (Chen *et al.*, 2023 ▸). eCDI is an advanced methodology base on CDI. In traditional CDI, only one sample can be illuminated by a totally coherent incident beam. In contrast, eCDI permits the illumination of multiple samples by a partially coherent incident beam while adhering to two additional constraints: (1) the sample must consist of ‘units’ that are identical in morphology and orientations, and (2) the unit size must fall within the coherent length of the incident beam and the distance between the units must exceed the coherent length of the incident beam. These constraints ensure that the diffraction patterns generated by each individual unit are identical and do not interfere with one another, resulting in what is termed an ensemble diffraction pattern. According to the principle of eCDI, if a sample comprises *N* such units, the diffraction pattern of the entire sample is equivalent to that of a single unit, but with the intensity magnified *N*×. Alternatively, eCDI can also be performed with totally coherent sources. In this case, interference fringes appear on the diffraction pattern from multiple units within the entire illuminated area. The presence of interference fringes depends on the arrangement of the units. These fringes can be mitigated by utilizing ensemble diffraction patterns from units that are arranged differently. Thus, the ensemble diffraction pattern approximates that of a single unit. Finally, the reconstruction processes of CDI and eCDI are identical.

**Figure 1:**
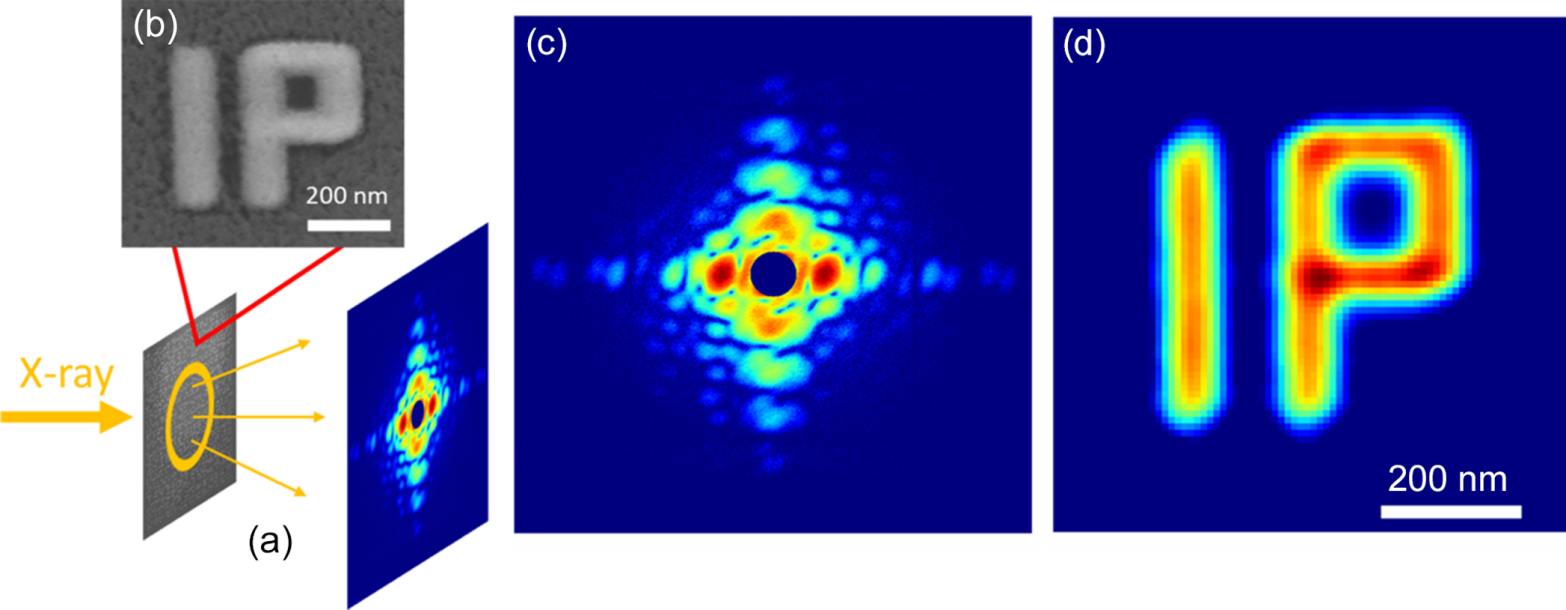
(*a*) Experimental setup for eCDI in which numerous unit patterns are randomly arranged on an SiN membrane and illuminated by coherent X-rays generating ensemble coherent scattering patterns. (*b*) eCDI unit pattern. (*c*) Collected ensemble coherent scattering pattern. (*d*) Reconstructed image.

Fig, 1 ▸(*a*) shows the ensemble CDI performed at TPS 13A and TPS 25A at the Taiwan Photon Source (TPS). The wavelengths of the two incident X-rays were approximately 1.4 Å. Eiger detectors 9M and 16M were located 6 m below the sample to collect the ensemble coherent scattering patterns. The intrinsic characteristics of the light sources at the two beamlines are distinct. TPS 13A is a partially coherent beamline with a beam size and coherent length of approximately 200 µm × 200 µm and 700 nm, respectively; TPS 25A is a nearly totally coherent beamline with a beam size of approximately 6 µm × 8 µm. The data collection processes and samples were carefully designed to satisfy the eCDI criteria described previously. In more detail, the sample comprised numerous unit patterns in ‘IP’ with a random arrangement, as shown in Fig. 1 ▸(*b*). The unit patterns were deposited with platinum and manufactured using the deposition mode of a focused ion beam (FIB) on a 500 nm-thick SiN membrane. The linewidth and the thickness of the ‘IP’ are both approximately 100 nm. The overall size of a single unit is about 450 nm and the spacing between units is around 1 µm, fulfilling the eCDI criteria for TPS 13A. Consequently, we captured an ensemble diffraction pattern directly in TPS 13A and 287 diffraction patterns at various illuminating positions in TPS 25A to compile an ensemble diffraction pattern. Fig. 1 ▸(*c*) shows the collected ensemble coherent scattering pattern. The patterns obtained at TPS 13A and TPS 25A were indistinguishable because they satisfied the ensemble criterion.

## 3. Data processing and image retrieval

The data processing and reconstruction algorithms are described as follows. A total of 2000 reconstructed results were obtained for each dataset using the same algorithm. Dead zones appeared on the detectors owing to the hardware limitations of the detectors and the beam stops used to protect them. To cover the region with missing data, the diffraction pattern was rotated by 180° to fill the missing data according to Friedel’s law, which states that the diffraction pattern is centrosymmetric for a non-absorbing sample (Vartanyants & Robinson, 2001 ▸). Datasets were cropped into 1201 × 1201 pixels for reconstruction, and the estimated pixel resolution of the reconstructed images calculated using the formula Δ*x* = λ*z*/*N*_p_Δ*p* was 9.2 nm, where Δ*x* is the real-space pixel size of the reconstructions, λ is the incident beam wavelength, *z* is the distance between the sample and the detector, *N*_p_ is the number of pixels used for the reconstruction, and Δ*p* is the pixel size of the detector (Burdet *et al.*, 2014 ▸). Finally, using the datasets prepared, the phase retrieval process is initiated using an iteration package, including hybrid input–output algorithm (HIO) (Fienup, 1982 ▸, 2013 ▸; Nishino *et al.*, 2003 ▸), dynamic support (Marchesini *et al.*, 2003 ▸) and error reduction (Fienup, 1982 ▸), where each reconstruction undergoes the iteration package three times; the iteration package includes: (1) update the supporting, (2) HIO 100 cycles, (3) ER 10 cycles and (4) HIO 20 cycles. Usually, ER is the final algorithmic step to truly investigate the depth of the local minimum HIO. However, during retrieval, the supports inducing the artificial signals on the boundary affect the retrieved images. Moreover, these artificial signals affect the result of FSC corresponding to the calculation for spatial resolution. To address the artificial signals, the last step in the iteration package is systematically set as HIO 20 cycles in this work. The diffraction intensity Fourier transform, which is the autocorrelation function of the object, estimates the HIO support in real space (Marchesini *et al.*, 2003 ▸; Fienup, 1982*a* ▸,*b* ▸, 2013 ▸).

**Figure 2:**
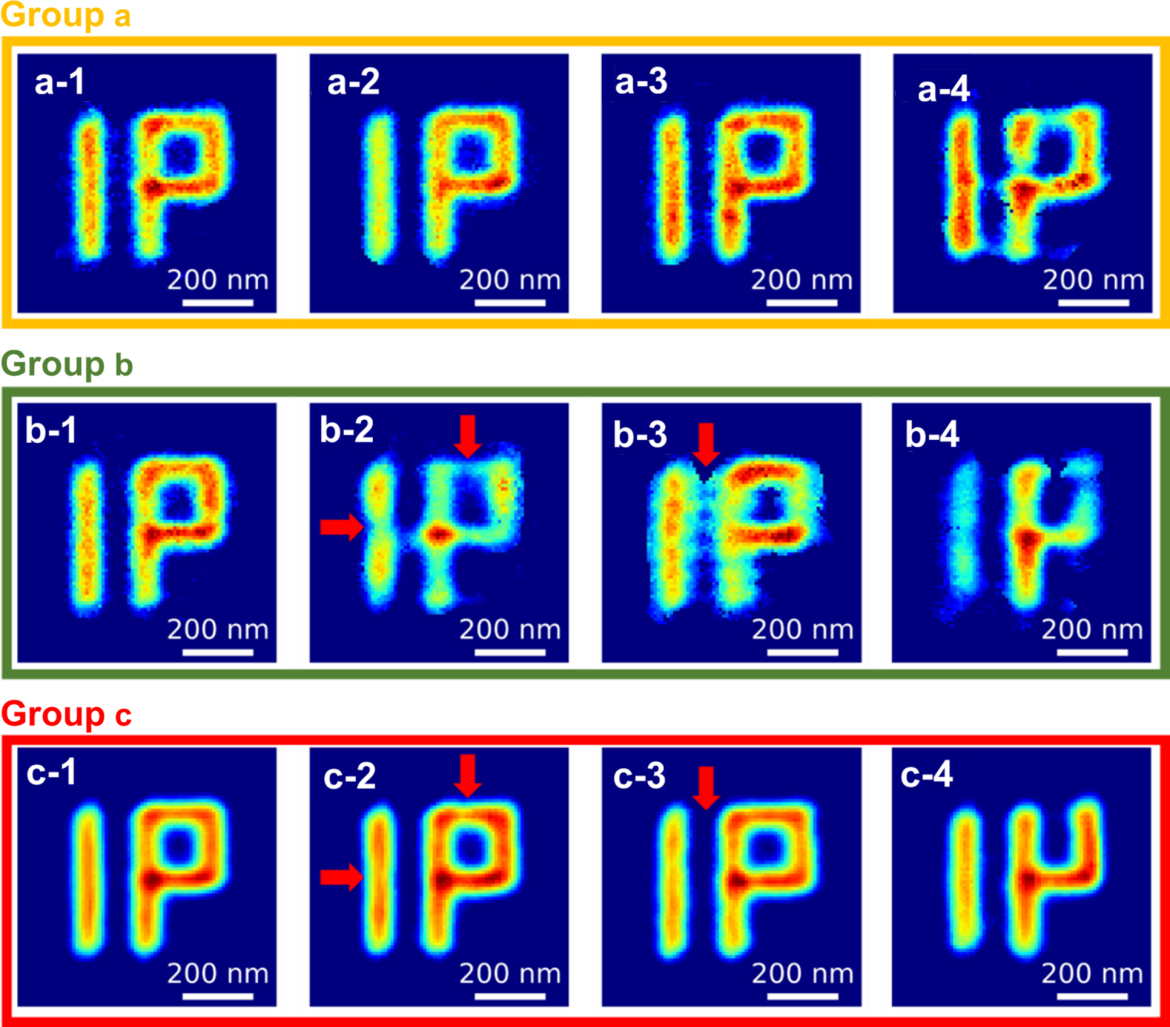
Group a: four images reconstructed using HIO within the top-10% reconstruction groups. Group b: four images within the top-10% reconstruction groups that appear unacceptable on visual inspection. Group c: Noise2Noise treated images corresponding to Group b. Note that all reconstructions are chosen by the indicator of Erf and start with random initial conditions.

**Figure 3:**
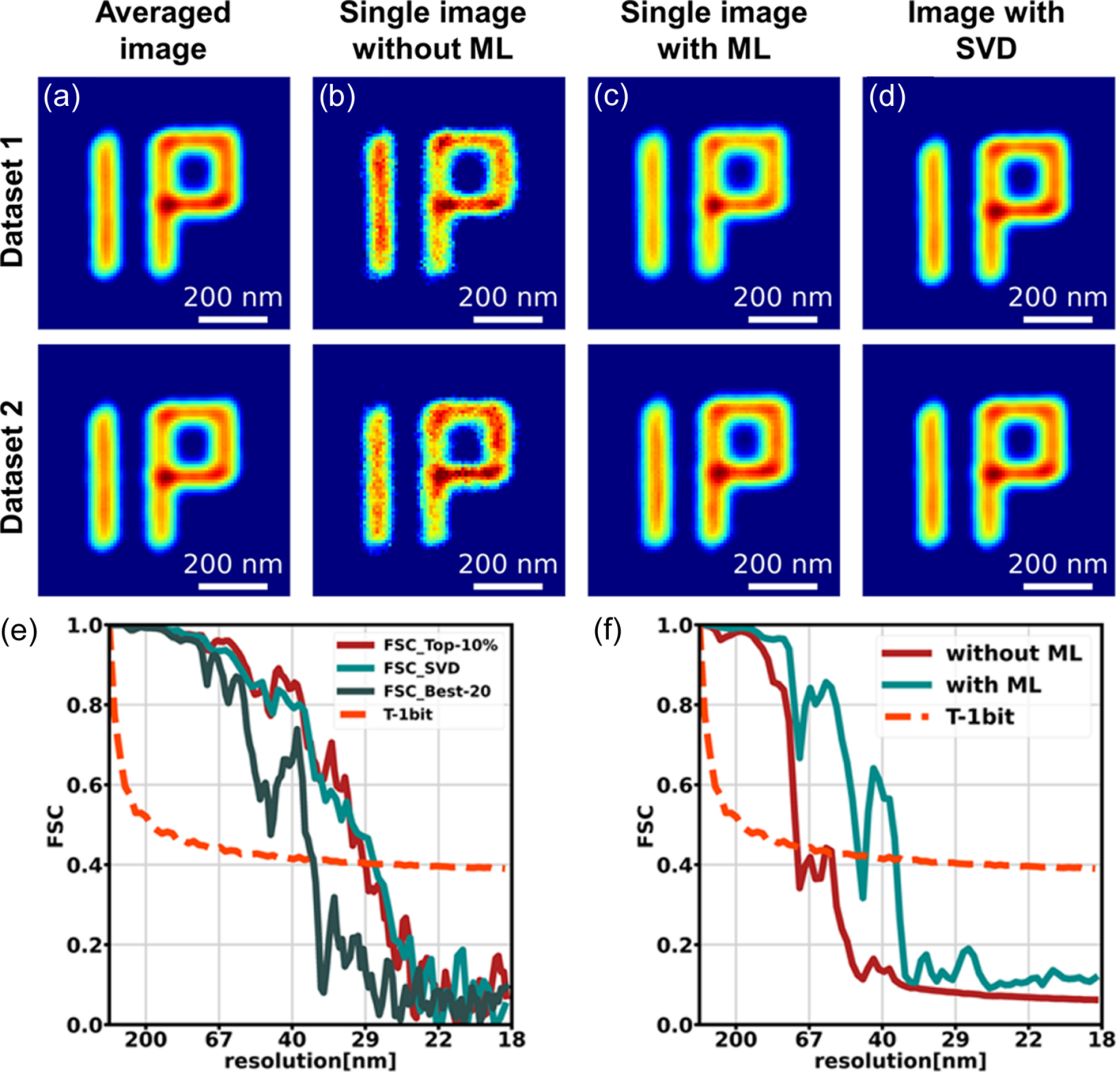
(*a*)–(*d*) Reconstructed images within the top-10% groups from the two datasets with different treatments for FSC calculations. (*a*) Average image of all reconstructed images. (*b*) Single reconstructed image from HIO. (*c*) Single denoised images of (*b*). (*d*) First eigen-solution by SVD analysis of all reconstructed images. The corresponding FSCs are shown in (*e*) and (*f*). For comparison, the FSC of the average of the best-20 reconstructions is shown in (*e*).

Two datasets with identical experimental conditions were subsequently acquired and prepared to estimate the spatial resolution using the Fourier shell correlation (FSC) method (Harauz & van Heel, 1986 ▸, 2005 ▸). Based on the previously described data processing method, 2000 reconstructions for each dataset were organized into three reconstruction groups using the error function (Erf) as the indicator of the degree of matching between the reconstructed and the measured scattering patterns. Accordingly, the 2000 reconstructions were categorized into three groups: the top 10% (200 reconstructions), the 10–40% (600 reconstructions) and the bottom 60% (1200 reconstructions). In this study, we focused on the top 10% and 10–40% groups and discarded the bottom-60% group. Four examples of reconstructed images in the top-10% are shown in Group a in Fig. 2 ▸. Compared with the scanning electron microscope (SEM) image of the unit pattern in Fig 1 ▸(*b*), defects and ambiguities, such as blurred edges, connections and disconnections, were present despite using the same phase retrieval processes. Based on multiple results, the reconstructed images can be averaged to obtain a single image to reduce defects. Fig. 3 ▸(*a*) shows the average image of the top-10% group. The quality of the averaged image was better than those in Group a in Fig. 2 ▸.

## 4. Denoising treatment

ML associated with image denoising was adopted to reduce the ambiguous features treated as noise in CDI-reconstructed images. ML algorithms are useful for performing nonlinear regression. Hence, image denoising was implemented using the Noise2Noise approach, with each 10–40% group from the two datasets divided into two stacks (Lehtinen *et al.*, 2018 ▸). Note that 10–40% groups using in ML is still extracted from the same 2000 reconstructed results. Consequently, a convolutional neural network was constructed and trained to project one stack onto another in the reconstruction domain. The identical information of both stacks was preserved, whereas the noise component did not regress owing to the lack of correlation between the two different reconstructions. Consequently, noise and defects in the reconstructions were reduced and a clearer image was acquired. This study focused on the applicability of the Noise2Noise approach for ambiguous features treated as noise in CDI-reconstructed images. The detailed mathematics and algorithms can be found in Lehtinen *et al.* (2018 ▸).

An open-source Python implementation associated with a mixed-scale dense (MS-D, https://dmpelt.github.io/msdnet) network architecture was employed (Pelt & Sethian, 2018 ▸; Pelt *et al.*, 2018 ▸; Flenner *et al.*, 2022 ▸). The two 10–40% groups (600 reconstructed images for each dataset) were selected to ensure sufficient training data. Initially, 600 reconstructed images with 151 × 151 pixels were divided into two stacks (300 reconstructed images per stack), one comprised the reconstructed input images and the other was used for training. A stack of the first 255 images was used for training, and the remaining 45 images were used for validation. Computations were performed on an NVidia Tesla P100 GPU running CUDA 10.2, and the network was tested for the top-10% groups. The stack of the tested images was different from that of the training images. The output of the tested images was computed using a Tesla P100 GPU. Generally, a common rule is to have at least thousands of images for training a dataset. Pelt & Sethian (2018 ▸) used hundreds of reconstructed images for training to denoise. In our work, hundreds of reconstructed images are used for training as well, which was sufficient for Noise2Noise to work. Note that in order to calculate a quantitative comparison of spatial resolution in the following section, both datasets with identical experimental conditions were trained separately.

Group b in Fig. 2 ▸ shows four images selected in the top-10% group. The reconstruction quality seems acceptable and unacceptable in b-1 and b-2 to b-4 in Fig. 2 ▸, respectively. The test and training images were different; however, ML associated with image denoising can significantly improve the quality of the reconstructed images. Group c in Fig. 2 ▸ represents the outputs of Group b in Fig. 2 ▸ after denoising, indicating a significant improvement in the image quality. Although b-2 and b-3 in Fig. 2 ▸ exhibit inferior image quality, the disconnection and connection corresponding to noise (red arrows) can be repaired and removed, as shown in c-2 and c-3. However, c-4 in Fig. 2 ▸ shows that the results exhibit inferior quality when the reconstructed image is severely damaged.

## 5. Discussion

A quantitative comparison of spatial resolution illustrates the denoising treatment behavior. To determine the spatial resolution, FSC was adopted as the indicator, and the threshold followed a T-1bit curve (Harauz & van Heel, 1986 ▸; van Heel & Schatz, 2005 ▸). The two reconstructed images from the two independent measurements were used to calculate the FSC, as indicated by datasets 1 and 2 in Fig. 3 ▸. The reconstructed images within the top-10% groups underwent the following four treatments: averaged images [Fig. 3 ▸(*a*)], one-to-one images [Fig. 3 ▸(*b*)], one-to-one images with denoising [Fig. 3 ▸(*c*)] and the first eigen-solution analysis (Favre-Nicolin *et al.*, 2020 ▸) [Fig. 3 ▸(*d*)].

The FSCs for an averaged image within the top 10% in Fig. 3 ▸(*a*) and the best 20 are shown in Fig. 3 ▸(*e*), where the spatial resolutions are 28.4 nm and 38.0 nm. The resolution of one-to-one images shown in Fig. 3 ▸(*b*), *i.e.* applying two individual reconstructed images from HIO directly to FSC calculation, was 75.1 nm; the red line in Fig. 3 ▸(*f*) denotes the corresponding FSC. The resolution of one-to-one images in Fig. 3 ▸(*b*) after undergoing denoising, as shown in Fig. 3 ▸(*c*), was 46.0 nm; the green line in Fig. 3 ▸(*f*) denotes the corresponding FSC. Compared with HIO images, the estimated resolution for ML was improved using FSC. Hence, ML can efficiently reduce ambiguous features treated as noise and extract consistent features from the reconstructed images. However, although ML eliminates the discrepancies and retains consistent signals, enhancing consistent artificial noise may disturb the reconstructions with ML. The reconstructions with poor quality and those with high resolution by ML are shown in Group b in Figs. 2 ▸ and 3 ▸(*c*), respectively. The resolution of an averaged image is higher than that of a reconstruction with ML, and the improvement of reconstruction for a ‘single’ image is demonstrated using FCS. Moreover, encountering a few unsuccessful results (*i.e.* c-4 in Fig. 2 ▸) is common in network based approaches, as observed in other fields that utilize ML.

On the other hand, the comparison given by Favre-Nicolin *et al.* (2020 ▸) with the approach demonstrated in our study is also shown. Top-40% data were used in the SVD method. The FSC for the image with the first eigen-solution in Fig. 3 ▸(*d*) is shown in Fig. 3 ▸(*e*). This result is very close to the averaged image and is greater than reconstructed images from direct HIO and ML results.

ML can potentially improve the quality of CDI reconstruction and offer advantages such as high-resolution output images from a trained network and fast computing from noisy to clear images (0.03 s). However, distinguishing the reconstructed image quality using ML and averaging remains challenging. The images that exhibit improved quality were defined by FSC in the spatial resolution. Evidently, the results reveal that, not only the highest resolution is an averaged HIO result, but the ML-denoised image shows improvement in the reconstruction. As indicated in the first paragraph of Section 4, the purpose of this work is the applicability of the Noise2Noise approach for ambiguous features treated as noise in CDI-reconstructed images. To our knowledge, a limitation of this approach to be used more commonly for a wide range of samples can be attributed to insufficient varieties of datasets corresponding to real samples. Note that when new samples are applied, the network should be re-trained.

## 6. Conclusions

This study focuses on reducing the ambiguity in CDI retrieval. The diffraction patterns applied in this research were adopted from an ensemble CDI measurement on an artificial test sample. The sample comprised numerous randomly arranged identical unit patterns, manufactured using the deposition mode of an FIB on an SiN membrane. Two datasets under identical experimental conditions were collected and 2000 reconstructed images were obtained from each dataset using the HIO method. The images selected for further analysis were determined based on the Erf metric of the reconstructed images. To diminish the ambiguity of images reconstructed with HIO, ML techniques associated with image denoising were employed. The Noise2Noise approach was used to perform image denoising, and a convolutional neural network was constructed and trained. An open-source implementation in Python, corresponding to a mixed-scale dense network architecture, was used to compare the images with and without denoising. The images reconstructed by ML exhibited improved quality. Additionally, eigen-solution analysis conducted by performing SVD on the same dataset was employed for comparison.

In addition to the demonstration of images, FSC was employed as an indicator to elucidate the impact of ML on image quality. Compared with the resolutions achieved by images reconstructed with HIO, the resolutions of the images reconstructed by ML were enhanced from 75.1 nm to 46.0 nm. These results highlight the efficiency of ML in reducing ambiguities and extracting consistent features from reconstructed images. Although the average and eigen-solution analysis presented better results, this work demonstrated the potential of the ML for CDI.

## Supplementary Material

Fig. S1. DOI: 10.1107/S1600577524006519/gy5061sup1.pdf
