# Supplementary material for: Using convolutional neural network denoising to reduce ambiguity in X-ray coherent diffraction imaging
Source: J Synchrotron Radiat. 2024 Aug 5;31(Pt 5):1340–5. doi: 10.1107/S1600577524006519 (PMC11371064; doi:10.1107/S1600577524006519)
Supplement: Supplementary file 1 [file s-31-01340-sup1.pdf]

JOURNAL OF SYNCHROTRON RADIATION

Volume 31 (2024)

Supporting information for article:

Using convolutional neural network denoising to reduce ambiguity in X-ray coherent diffraction imaging

Kang-Ching Chu, Chia-Hui Yeh, Jhih-Min Lin, Chun-Yu Chen, Chi-Yuan Cheng, Yi-Qi Yeh, Yu-Shan Huang and Yi-Wei Tsai

Machine learning (ML) associated with image denoising was adopted to reduce the ambiguous features treated as noise in reconstructions of coherent diffraction imaging (CDI). Both two datasets with identical experimental conditions were trained separately for each dataset. Note that 10-40% groups (600 reconstructions) using in ML was extracted from a dataset with 2000 reconstructions, which calculated from the same CDI algorithm. The experiments were performed with mixed-scale dense (MS-D) networks, which were trained for 2000 epochs with the learning rate 0.001. The mean-squared error between output and target images was adopted as an indicator to improve ML networks. Figure S1 shows the variation of normalized error with epochs for both two datasets. It is clearly to seen that the error is decreased dramatically before 250 epochs and then becomes smooth until 2000 epochs indicating the minimization for iterations.

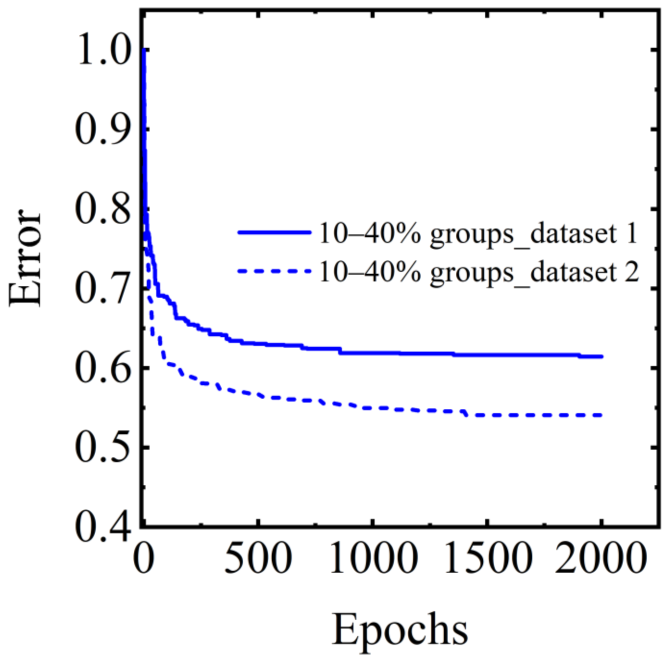

Figure S1. The normalized error of the validation set as training progressed with the learning rate = 0.001.
